# Central retinal artery occlusion following surgery for thyroid eye disease: A case report

**DOI:** 10.1097/MD.0000000000040283

**Published:** 2024-11-15

**Authors:** Baozhu Dai, Kaiming Gu, Feng Tan, Suhui Zhu, Yan Dai

**Affiliations:** a Department of Ophthalmology, Mianyang Central Hospital, Mianyang, Sichuan, China.

**Keywords:** central retinal artery occlusion, orbital decompression, thyroid eye disease

## Abstract

**Rationale:**

Thyroid eye disease (TED) is the most common orbital disorder in adults and significantly affects patient health. Orbital decompression surgery is an important treatment option. Central retinal artery occlusion (CRAO) after orbital medial wall decompression is rare in patients with TED. Therefore, the earlier the identification and treatment, the more likely it is to reduce visual impairment.

**Patient concerns:**

This paper examines a case of CRAO occurring postoperatively in a patient who underwent medial wall orbital decompression for TED.

**Diagnoses:**

Central retinal artery occlusion.

**Interventions:**

During the operation, the pupil was dilated, and eye massage and peribulbal injection of atropine were performed immediately. Fundus fluorescein angiography suggested the possibility of CRAO. Intravenous methylprednisolone 1000 mg, mannitol 50 g, ginkgo biloba extract 20 mL, nimodipine 20 mg tid, cobamamide 0.5 mg tid, and oral citicoline 0.2 g tid, along with periocular injection of atropine and hyperbaric oxygen therapy were also administered.

**Outcomes:**

Fifteen days after onset, the patient’s retinal edema and retinal blood perfusion greatly improved. The patient’s visual acuity recovered from counting fingers to 0.6.

**Lessons:**

Retinal vascular obstruction is a serious threat to vision; therefore, early detection and treatment are very important.

## 1. Introduction

Thyroid associated ophthalmopathy, also known as thyroid eye disease (TED), is the most prevalent orbital disorder in adults.^[1]^ The primary clinical manifestations include eyelid retraction, proptosis, and restrictive strabismus, among other symptoms. In severe cases, these symptoms may lead to exposure keratopathy and optic neuropathy. Orbital decompression surgery is an essential treatment for thyroid associated ophthalmopathy patients. With the utilization of endoscopy, methods for orbital decompression include endoscopic medial wall, lateral wall, and floor decompression, as well as multiwall combined surgeries. However, endoscopic orbital decompression is challenging, due to limited operative space and visual field. Additionally, the intricate anatomy within the orbit poses risks for severe complications such as damage to the nasolacrimal duct, sinusitis, cerebrospinal fluid leaks, changes in pupil size, vision impairment, intracranial infection, and vascular injuries.^[2,3]^ This paper reports and analyzes a case in which a patient undergoing orbital decompression experienced pupil dilation and was later diagnosed with central retinal artery occlusion (CRAO) postoperatively.

## 2. Case presentation

The patient was a 27-year-old female with a history of myopia in both eyes for over 10 years and underwent bilateral blepharoplasty 6 years ago. She was diagnosed with hyperthyroidism 2 years prior, which led to hypothyroidism following Iodine-131 treatment. The patient had previously undergone radiation therapy for both eyes 10 times. She underwent successful “endoscopic right orbital decompression” in our department half a month ago and was admitted for the planned surgery on the left eye.

**Figure 1.**
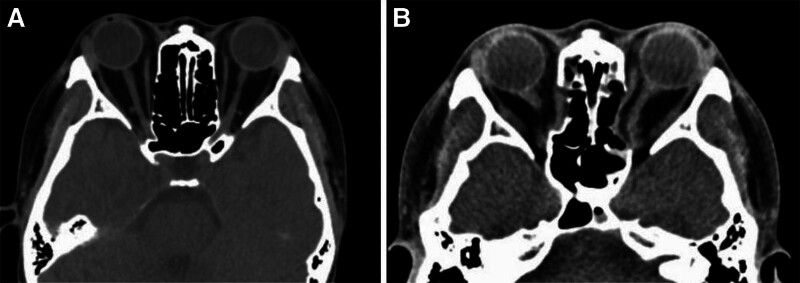
The results of preoperative and postoperative orbital CT. Preoperative orbital CT showed the left eye was obviously convex and the right extraneous eye decreased (A). Postoperative orbital CT showed the changes after bilateral orbital decompression (B). CT = computerized tomography.

Ophthalmological examination upon admission:

Visual acuity: OD (oculus dexter): corrected to 1.0; OS (oculus sinister): corrected to 1.0.
Cornea: transparent in the right eye, partially epithelial staining in the left, otherwise transparent.
Anterior chamber depth: normal in both eyes.
Pupil: round, approximately 2.5 mm, with adequate light reflex.
Lens: transparent.
Vitreous: no significant opacities.
Retina: flat, optic disc pale.
Intraocular pressure: right 15 mm Hg, left 14 mm Hg.
Lacrimal sac area: no purulent discharge upon pressure.
Eye movement: OD: limited outward rotation; OS: slightly limited outward rotation.
Ocular alignment: 33 cm ou cc 2^△^BO.
Exophthalmometry: OD: 14 mm; OS: 17 mm.
Orbital computerized tomography: The left eye was obviously convex, and Graves ophthalmopathy was considered. The right orbital changed after the operation, and the right extraneous eye decreased (Fig. 1A).
Admission diagnosis:
  1. Bilateral TED
  2. Bilateral myopia
  3. Bilateral corneal astigmatism
  4. Secondary hypothyroidism

**Figure 2.**
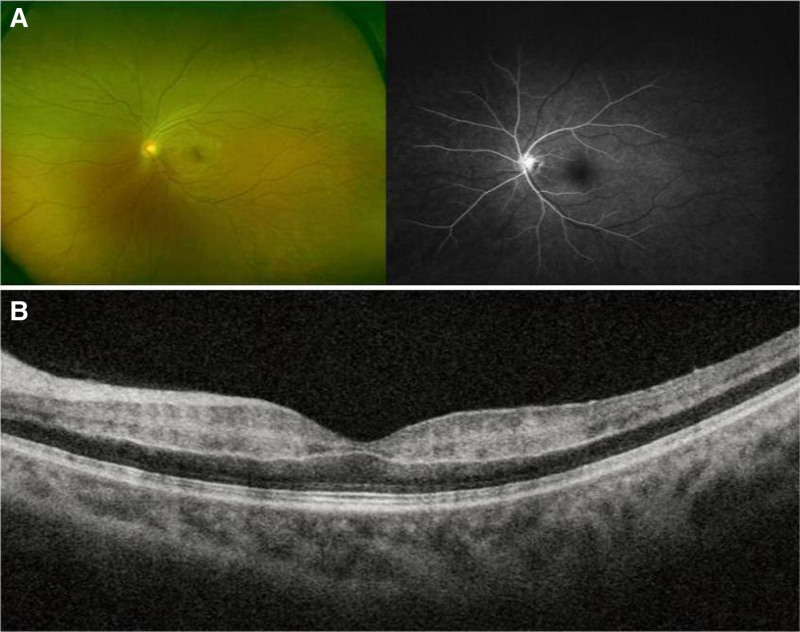
Results of fundus examination on the first day after orbital decompression. Fundus fluorescein angiography showed delayed arterial filling, prominent “front” phenomenon, strong fluorescence of the optic disc, and non-perfusion of the retinal capillaries (A). Optical coherence tomography revealed swelling and structural blurring of the retina (B).

**Figure 3.**
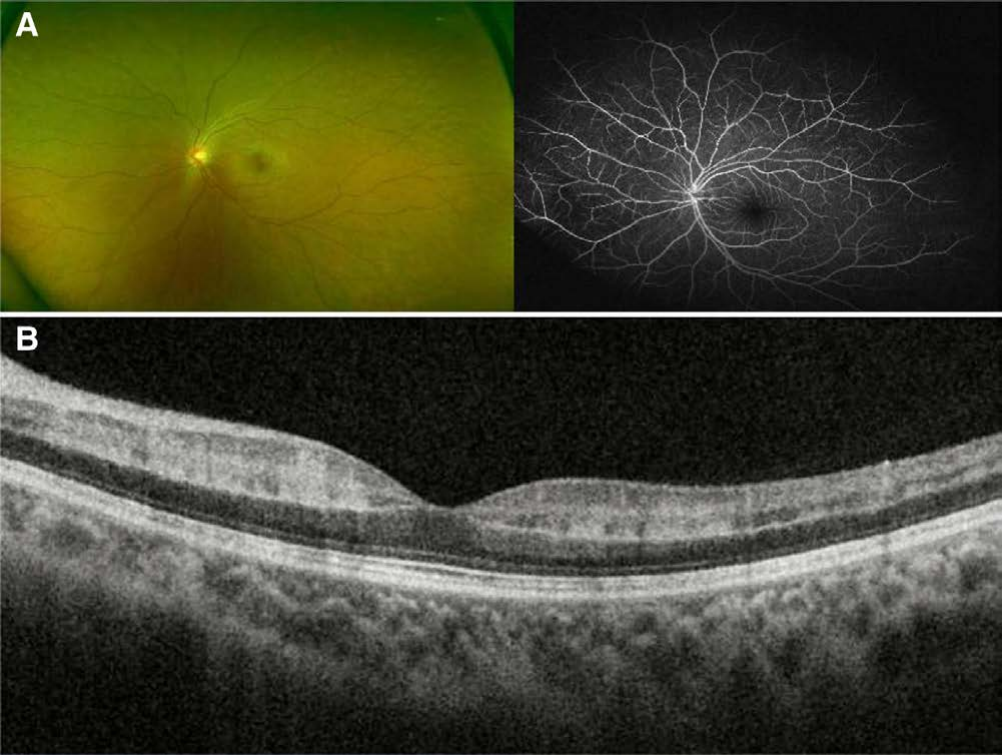
The results of fundus examination on the 15th day after orbital decompression. Fundus fluorescein angiography showed that retinal edema and retinal blood perfusion were significantly improved (A). Optical coherence tomography revealed great improvement of the retinal edema and retinal blood perfusion (B).

After completing preoperative assessments, the patient underwent “endoscopic left orbital decompression.” During the procedure, pupil dilation of approximately 8 mm was observed during the removal of intraconal fat from the medial wall. Immediate eyeball massage and periocular injection of atropine were administered. Postoperative treatment included intravenous administration of 1000 mg methylprednisolone and mannitol. On the first postoperative day, visual acuity was counting fingers at 30 cm. Subsequently, fundus fluorescein angiography showed delayed arterial filling, prominent “front” phenomenon, strong fluorescence of the optic disc, and non-perfusion of the retinal capillaries (Fig. 2A), suggesting the possibility of CRAO. In addition, optical coherence tomography showed the retinal edema and structural ambiguity (Fig. 2B). The following medications and treatments were administered: intravenous methylprednisolone 1000 mg, ginkgo biloba extract 20 mL, nimodipine 20 mg tid, cobamamide 0.5 mg tid, and oral citicoline 0.2 g tid, along with periocular injection of atropine and hyperbaric oxygen therapy. Two weeks later, the patient’s retinal edema and retinal blood perfusion greatly improved (Fig. 3A and B), and the visual acuity improved to 0.6.

## 3. Discussion

CRAO is a severe, vision-threatening ophthalmic emergency that requires immediate intervention. Timely and effective treatment measures are critical for the recovery of vision in affected patients. In this case, the patient experienced pupil dilation intraoperatively, and postoperative fundus fluorescein angiography showed delayed arterial filling, a prominent “front” phenomenon, strong fluorescence of the optic disc, and non-perfusion of the retinal capillaries, suggesting the possibility of CRAO. Further computerized tomography examination revealed a noticeable shift of the medial rectus muscle and optic nerve towards the medial wall, along with optic nerve swelling, indicating potential damage to the intraorbital segment of the optic nerve and CRAO (Fig. 1B). Given the swollen extraocular muscles and orbital soft tissues in patients with TED, the volume of the orbit increases, reducing orbital compliance and leading to elevated orbital pressure. The impedance in orbital venous return further raises episcleral venous pressure, subsequently increasing intraocular pressure.^[4]^ The sudden drop in orbital pressure after opening the medial orbital wall and removing some orbital fat may have caused a significant shift of the optic nerve along with the medial rectus muscle, leading to potential damage to the intraorbital segment of the optic nerve. Simultaneously, the abrupt change in pressure may have induced spasms in the central retinal artery, causing CRAO. Current treatments for CRAO include both traditional conservative treatments and unconventional therapies. Traditional treatments mainly involve lowering intraocular pressure to reduce arterial perfusion resistance, inhaling a mixture of 95% oxygen and 5% carbon dioxide to alleviate retinal hypoxia and dilate blood vessels, and the use of vasodilators, thrombolytics, and antiplatelet agents. Nontraditional treatments include super-selective ophthalmic artery thrombolysis, laser embolysis, subfascial collagen sponge infusion, and stellate ganglion block. Although distal microcatheterization of the ophthalmic artery is feasible, it increases the risk of arterial dissection and thromboembolic events.^[5]^ In this case, conservative treatment was opted for, considering the potential for both CRAO and damage to the intraorbital segment of the optic nerve.

For patients with moderate to severe proptosis, we are also contemplating the necessity of gradient decompression. On the one hand, gradient decompression can reduce postoperative inflammatory responses. On the other hand, this technique allows for controlled removal of intraconal fat, leading to a more gradual change in orbital pressure and reducing the likelihood of damage to the optic nerve and intraocular arteries. Recent literature suggests that preserving some of the strut structures and maintaining the periorbita on the medial wall can reduce postoperative diplopia. Although this patient had no diplopia postoperatively, there was a noticeable shift in the medial rectus muscle and the optic nerve. Hence, the possibility of reducing such shifts by preserving some of the strut structures and the periorbita should be considered.^[6,7]^ During endoscopic orbital decompression surgery, the operative eye should not be covered so that any abnormal pupil constriction or dilation can be immediately observed. If such a change occurs, the surgery should be halted for emergency intervention. If necessary, real-time fundus examination can be conducted intraoperatively. If blurred optic disc margins, a cherry-red spot in the macular area, or thinning of central arteries and veins are observed, immediate treatment for CRAO should be initiated. The earlier the treatment, the better the outcome. Immediate eyeball massage, anterior chamber paracentesis, oxygen inhalation, systemic corticosteroids, and nerve nutrition therapies should be administered to salvage the patient’s vision as much as possible.

## 4. Conclusion

This case report demonstrates that gradient decompression may be required during endoscopic orbital decompression. In addition, the changes of the pupil of the operative eye should be observed during the operation, and any abnormalities must be addressed immediately. Finally, early diagnosis and treatment are critical for embolic retinal vascular occlusion.

## Acknowledgments

The authors thank the patient who participated in the present study for providing written permission to publish this case report.

## Author contributions

**Conceptualization:** Baozhu Dai, Kaiming Gu, Yan Dai.

**Data curation:** Baozhu Dai, Kaiming Gu, Feng Tan, Suhui Zhu.

**Formal analysis:** Baozhu Dai, Kaiming Gu, Yan Dai.

**Writing – original draft:** Baozhu Dai.

**Writing – review & editing:** Kaiming Gu, Yan Dai.
